# 639. Are SARS-CoV-2 antigenemia measurements still relevant? Comparison of Omicron era to early pandemic plasma nucleocapsid levels

**DOI:** 10.1093/ofid/ofad500.703

**Published:** 2023-11-27

**Authors:** Gregory L Damhorst, Hans Verkerke, Sydney E Martin, Eli Wilber, Wilbur A Lam

**Affiliations:** Emory University, Atlanta, Georgia; Emory University School of Medicine, Atlanta, Georgia; Emory University, Atlanta, Georgia; Emory University School of Medicine, Atlanta, Georgia; Emory University School of Medicine/Georgia Institute of Technology, Atlanta, GA

## Abstract

**Background:**

Viral nucleocapsid in the blood (antigenemia) is a sensitive marker of acute COVID-19 and is supported in numerous published studies to have diagnostic and prognostic value. Review of the literature shows that nearly all antigenemia studies published as of February 25, 2023 precede the emergence of the Delta and Omicron variants. Whether the observations made in early pandemic literature can be applied to modern variants is unknown.

Timeline of the SARS-CoV-2 antigenemia literature highlights the paucity of Omicron-era data

**Figure:**
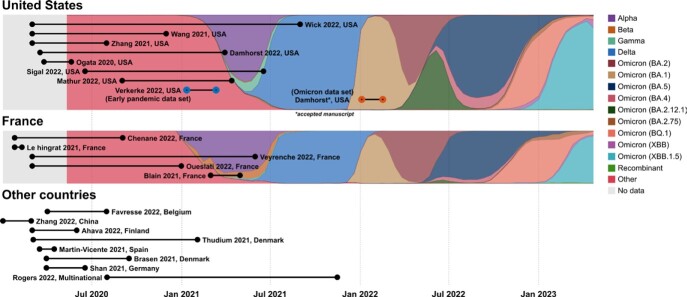

Sampling periods described in published studies of SARS-CoV-2 antigenemia identified through a systematic review of search results from PubMed, Embase, and Scopus (conducted February 25, 2023) overlayed on variant proportion charts (from GISAID via covariants.org and ourworldindata.org). Although more than 15 months had passed since the emergence of the Omicron variant, no studies were yet published from this timeframe.

**Methods:**

Convenience samplings of residual blood specimens from routine clinical care were collected at Emory Healthcare in Atlanta, GA. Plasma nucleocapsid protein measurements were performed using the Quanterix Simoa ultrasensitive immunoassay platform. Cohort characteristics are described in depth in prior publications (Verkerke, et al. 2022 and an accepted manuscript by Damhorst, et al.). We performed a new analysis of plasma nucleocapsid levels comparing the early pandemic (Verkerke) data set and the Omicron (Damhorst) data set. We examined overall distributions of antigenemia in both data sets. We then estimated 95% distribution boundaries from early pandemic data stratified by duration of symptoms and compared these to point measurements of Omicron antigenemia.

**Results:**

Median antigenemia levels for early pandemic (N = 244) and Omicron (N =31) data were 88.4 pg/mL (IQR 9.9-1235.5) and 137.1 pg/mL (IQR 14.8-368.5), respectively. There was no significant difference by rank sum test (p = 0.97) between the two cohorts. Stratification by days of symptoms at time of sampling showed that 16 of 19 (84%) Omicron measurements fell within the 95% distribution estimate determined from early pandemic data, but 13 of 19 fell below the median trend line.

Omicron era SARS-CoV-2 antigenemia samples follow a distribution similar to early pandemic samples

**Figure:**
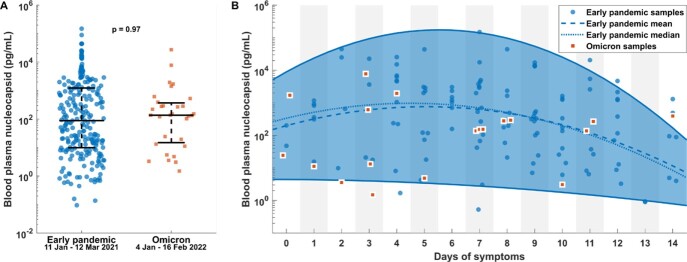

(A) Blood nucleocapsid levels measured during the early pandemic compared to the Omicron era exhibited similar median values and were not significantly different by rank sum test. (B) Data binned by days since symptom onset. Mean, median and standard deviation (SD) of the log antigenemia level were calculated from early pandemic data in each bin. Quadratic regressions were calculated to fit mean, median and mean+/-1.96*SD versus time which defined the boundaries of a 95% distribution interval. Omicron era measurements were overlaid suggesting they follow a similar distribution pattern but may fall lower than the mean and median of early pandemic levels.

**Conclusion:**

There are multiple potential applications for SARS-CoV-2 nucleocapsid measurements, but there is a paucity of published data since the emergence of the Omicron variant. Our data suggest that Omicron antigenemia follows a similar overall distribution but when stratified by symptom duration may indicate lower absolute antigenemia levels compared to the early pandemic. More data are needed, and future studies should aim to validate clinical uses of antigenemia measurements with modern variants.

**Disclosures:**

**All Authors**: No reported disclosures

